# Supplementary material for: Safety outcomes of ticagrelor among patients with STE-ACS post streptokinase therapy-a retrospective observational study
Source: PLoS One. 2023 Aug 4;18(8):e0289721. doi: 10.1371/journal.pone.0289721 (PMC10403104; doi:10.1371/journal.pone.0289721)
Supplement: S1 Checklist — (PDF) [file pone.0289721.s001.pdf]

STROBE Statement—checklist of items that should be included in reports of observational studies

| | Item No. | Recommendation | Page No. | Relevant text from manuscript |
|---|---|---|---|---|
| **Title and abstract** | 1 | (*a*) Indicate the study's design with a commonly used term in the title or the abstract | 1 | line number: 1-2 |
| | | (*b*) Provide in the abstract an informative and balanced summary of what was done and what was found | 1-2 | line number: 16 (page 1)-36 (page 2) |
| **Introduction** | | | | |
| Background/rationale | 2 | Explain the scientific background and rationale for the investigation being reported | 3 | line number: 39-48 |
| Objectives | 3 | State specific objectives, including any prespecified hypotheses | 4 | line number: 64-67 |
| **Methods** | | | | |
| Study design | 4 | Present key elements of study design early in the paper | 4 | line number: 70 |
| Setting | 5 | Describe the setting, locations, and relevant dates, including periods of recruitment, exposure, follow-up, and data collection | 4 | line number: 70-73 |
| Participants | 6 | (*a*) *Cohort study*—Give the eligibility criteria, and the sources and methods of selection of participants. Describe methods of follow-up<br>*Case-control study*—Give the eligibility criteria, and the sources and methods of case ascertainment and control selection. Give the rationale for the choice of cases and controls<br>*Cross-sectional study*—Give the eligibility criteria, and the sources and methods of selection of participants | 4 to 5 | line number: 78 (page 4) -83 (page 5) |
| | | (*b*) *Cohort study*—For matched studies, give matching criteria and number of exposed and unexposed<br>*Case-control study*—For matched studies, give matching criteria and the number of controls per case | N/A | no matching |
| Variables | 7 | Clearly define all outcomes, exposures, predictors, potential confounders, and effect modifiers. Give diagnostic criteria, if applicable | 5 | line number: 88-91 |
| Data sources/ measurement | 8* | For each variable of interest, give sources of data and details of methods of assessment (measurement). Describe comparability of assessment methods if there is more than one group | 5 | line number: 92 |
| Bias | 9 | Describe any efforts to address potential sources of bias | 5-6 | line number: 99 (page 5)-100 (page6) |
| Study size | 10 | Explain how the study size was arrived at | 4-5 | line number: 78 (page4)-80 (page5) |

| | | | | |
|---|---|---|---|---|
| Quantitative variables | 11 | Explain how quantitative variables were handled in the analyses. If applicable, describe which groupings were chosen and why | 11 | line number: 164 |
| Statistical methods | 12 | (*a*) Describe all statistical methods, including those used to control for confounding | 5-6 | line number: 99 (page 5)-101 (page 6) |
| | | (*b*) Describe any methods used to examine subgroups and interactions | N/A | no subgroup analysis |
| | | (*c*) Explain how missing data were addressed | N/A | no missing data |
| | | (*d*) *Cohort study*—If applicable, explain how loss to follow-up was addressed<br>*Case-control study*—If applicable, explain how matching of cases and controls was addressed<br>*Cross-sectional study*—If applicable, describe analytical methods taking account of sampling strategy | N/A | no patient loss to follow-up |
| | | (*e*) Describe any sensitivity analyses | N/A | no sensitivity analysis |
| **Results** | | | | |
| Participants | 13* | (a) Report numbers of individuals at each stage of study—eg numbers potentially eligible, examined for eligibility, confirmed eligible, included in the study, completing follow-up, and analysed | 6 | line number: 109-111 |
| | | (b) Give reasons for non-participation at each stage | in Fig 1 | Fig 1 |
| | | (c) Consider use of a flow diagram | 6 | line number: 111 (Fig 1) |
| Descriptive data | 14* | (a) Give characteristics of study participants (eg demographic, clinical, social) and information on exposures and potential confounders | 7 | table 1 |
| | | (b) Indicate number of participants with missing data for each variable of interest | N/A | no missing variable of interest |
| | | (c) *Cohort study*—Summarise follow-up time (eg, average and total amount) | 6 | line number: 112 |
| Outcome data | 15* | *Cohort study*—Report numbers of outcome events or summary measures over time | 9 | line number: 141-142 |
| | | *Case-control study*—Report numbers in each exposure category, or summary measures of exposure | N/A | N/A |
| | | *Cross-sectional study*—Report numbers of outcome events or summary measures | N/A | N/A |
| Main results | 16 | (*a*) Give unadjusted estimates and, if applicable, confounder-adjusted estimates and their precision (eg, 95% confidence interval). Make clear which confounders were adjusted for and why they were included | 11 | table 3 |
| | | (*b*) Report category boundaries when continuous variables were categorized | 10 | table 2 (hemoglobin, eGFR) |
| | | (*c*) If relevant, consider translating estimates of relative risk into absolute risk for a meaningful time period | N/A | N/A |

Continued on next page

| | | | | |
|---|---|---|---|---|
| Other analyses | 17 | Report other analyses done—eg analyses of subgroups and interactions, and sensitivity analyses | 10, 12 | table 2 (variables associated with $P2Y_{12}$ inhibitors switching)<br>table 3 (efficacy outcome as secondary outcome) |
| **Discussion** | | | | |
| Key results | 18 | Summarise key results with reference to study objectives | 13-14 | line number: 186 (page 13) and 211 (page 14) |
| Limitations | 19 | Discuss limitations of the study, taking into account sources of potential bias or imprecision. Discuss both direction and magnitude of any potential bias | 15-16 | line number: 243 (page 15)-246 (page 16) |
| Interpretation | 20 | Give a cautious overall interpretation of results considering objectives, limitations, multiplicity of analyses, results from similar studies, and other relevant evidence | 13-15 | line number: 188 (page 13)-242 (page 15) |
| Generalisability | 21 | Discuss the generalisability (external validity) of the study results | 16 | line number: 248-252 |
| **Other information** | | | | |
| Funding | 22 | Give the source of funding and the role of the funders for the present study and, if applicable, for the original study on which the present article is based | in submission form | in submission form |

*Give information separately for cases and controls in case-control studies and, if applicable, for exposed and unexposed groups in cohort and cross-sectional studies.

**Note:** An Explanation and Elaboration article discusses each checklist item and gives methodological background and published examples of transparent reporting. The STROBE checklist is best used in conjunction with this article (freely available on the Web sites of PLoS Medicine at http://www.plosmedicine.org/, Annals of Internal Medicine at http://www.annals.org/, and Epidemiology at http://www.epidem.com/). Information on the STROBE Initiative is available at www.strobe-statement.org.
