# Supplementary material for: Safety outcomes of ticagrelor among patients with STE-ACS post streptokinase therapy-a retrospective observational study
Source: PLoS One. 2023 Aug 4;18(8):e0289721. doi: 10.1371/journal.pone.0289721 (PMC10403104; doi:10.1371/journal.pone.0289721)
Supplement: S1 Fig — (PDF) [file pone.0289721.s002.pdf]

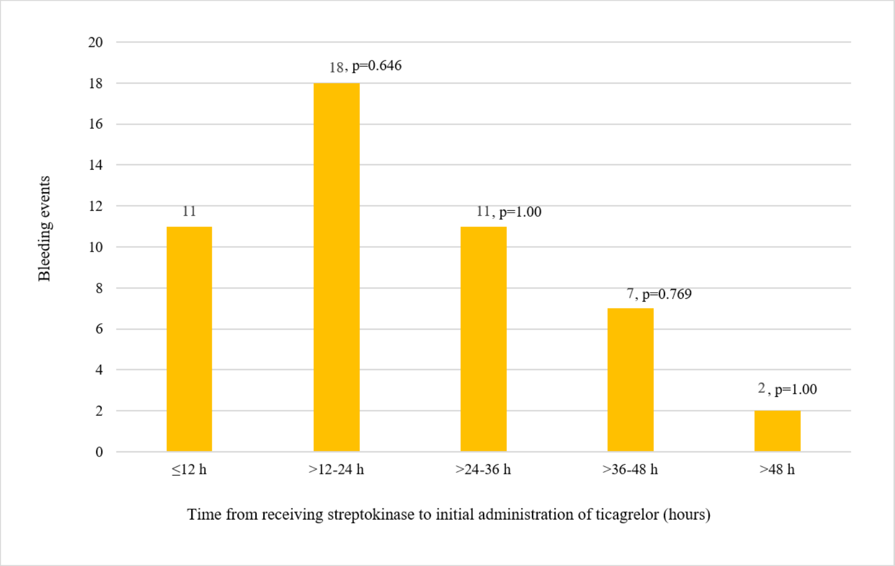

S1 Fig. Bleeding events and time from streptokinase to ticagrelor initiation in the switch group, particularly bleeding events post switching. p-value compared between switching within 12 hours and other times.
