# Supplementary material for: Safety outcomes of ticagrelor among patients with STE-ACS post streptokinase therapy-a retrospective observational study
Source: PLoS One. 2023 Aug 4;18(8):e0289721. doi: 10.1371/journal.pone.0289721 (PMC10403104; doi:10.1371/journal.pone.0289721)
Supplement: S1 Table — (PDF) [file pone.0289721.s003.pdf]

S1 Table. Procedures and other baseline characteristics.

Characteristic	All (n=401)	Switch (n=199)	No switch (n=202)	p-value[1]
**Streptokinase dose, n (%)**				
1.5 million units	162 (40.40)	56 (28.14)	106 (52.4)	<0.001
0.75 million units	239 (69.60)	143 (71.86)	96 (47.52)	
**Clopidogrel loading dose, n (%)**				
300 mg	353 (88.02)	175 (87.94)	178 (88.11)	0.470
>300 mg	9 (2.24)	4 (2.01)	5 (2.48)	1.000
no loading (75 mg)	33 (8.23)	14 (7.04)	19 (9.41)	-
**Pre-PCI medications, n (%)**				
Enoxaparin	201 (50.12)	98 (49.25)	103 (50.99)	0.615
Fondaparinux	6 (1.50)	1 (0.50)	5 (2.48)	0.117
**Peri-PCI medications, n (%)**				
Unfractionated heparin	206 (51.37)	94 (47.24)	112 (55.45)	1.000
Enoxaparin	133 (33.17)	77 (38.69)	56 (27.72)	0.123
Glycoprotein IIb/IIIa inhibitors	54 (13.47)	26 (13.07)	28 (13.86)	0.884
**Discharge medications, n (%)**				
Aspirin	390 (97.26)	196 (98.49)	194 (96.04)	0.220
Clopidogrel	202 (50.37)	8 (4.02)	194 (96.04)	<0.001
ACEis	223 (55.61)	118 (59.30)	105 (51.98)	0.160
ARBs	22 (5.49)	15 (7.54)	7 (3.47)	0.082
MRAs	36 (8.98)	15 (7.54)	21 (10.40)	0.383
Beta-blockers	214 (53.37)	100 (50.25)	114 (56.44)	0.230
Statins	389 (97.10)	196 (98.49)	193 (95.54)	0.140
PPIs	298 (74.31)	140 (70.35)	158 (78.22)	0.086
**In-hospital procedures, n (%)**				
Femoral access	306 (76.3)	164 (82.41)	142 (70.30)	0.005
Number of lesions treated, mean±SD	1.2±0.5	1.2±0.5	1.2±0.5	0.524
Number of stents implanted, median (IQR)	1 (1-2)	1 (1-2)	1 (1-2)	0.802
intravascular imaging	41 (10.22)	32 (16.08)	9 (4.46)	<0.001
IVUS	28 (6.98)	20 (10.05)	8 (3.96)	-
OCT	14 (3.49)	12 (6.03)	2 (0.99)	-
**Bleeding site**				
Bleeding event, n	166	83	83	-
PCI access site, n (%)	94 (56.63)	50 (60.24)	44 (53.01)	0.543
GI hemorrhage, n (%)	31 (18.67)	12 (14.46)	19 (22.89)	0.438
Others, n (%)	41 (24.70)	21 (25.30)	20 (24.10)	0.740

[1]without IPW adjusted; PCI, percutaneous coronary intervention; SD, standard deviation; IQR, interquartile range; ACEis, Angiotensin-converting enzyme inhibitors; ARBs, Angiotensin II Receptor Blockers; MRAs, Mineralocorticoid Receptor Antagonists; PPIs, Proton pump inhibitors; IVUS, intravascular ultrasound; OCT, Optical Coherence Tomography; GI, gastrointestinal
