# Supplementary material for: Safety outcomes of ticagrelor among patients with STE-ACS post streptokinase therapy-a retrospective observational study
Source: PLoS One. 2023 Aug 4;18(8):e0289721. doi: 10.1371/journal.pone.0289721 (PMC10403104; doi:10.1371/journal.pone.0289721)
Supplement: S2 Table — (PDF) [file pone.0289721.s004.pdf]

S2 Table. Standardized difference of baseline characteristics with and without adjusted IPW.

Characteristics	Standardized difference without IPW adjusted	Standardized difference with IPW adjusted
Age	-0.240	-0.015
Sex	-0.123	0.020
Body weight	0.244	-0.041
hemoglobin	0.191	-0.045
HT	-0.089	0.007
DLP	0.073	-0.051
DM	0.124	0.005
CAD	0.487	0.032
Stroke, TIA	0.040	0.006
eGFR	0.279	-0.031
streptokinase to PCI	-0.149	-0.051
PCI strategy	0.172	-0.051
Anticoagulant administration Pre -PCI	0.074	0.033
Streptokinase dose	0.511	-0.001
Glycoprotein IIb/IIIa inhibitor	-0.023	-0.036
PCI access site	-0.287	0.095
Number of lesion treated	-0.082	0.032
Number of coronary stent	0.003	0.032
intravascular imaging	0.240	-0.079

HT, hypertension; DLP, dyslipidemia; DM, diabetes mellitus; CAD; cardiovascular disease; TIA, transient ischemic attack; IQR, interquartile range; PCI, percutaneous coronary intervention; IQR, interquartile range.
